# Comparison of cardiovascular biomarker expression in extracellular vesicles, plasma and carotid plaque for the prediction of MACE in CEA patients

**DOI:** 10.1038/s41598-023-27916-6

**Published:** 2023-01-18

**Authors:** Maarten C. Verwer, Joost Mekke, Nathalie Timmerman, Farahnaz Waissi, Arjan Boltjes, Gerard Pasterkamp, Gert J. de Borst, Dominique P. V. de Kleijn

**Affiliations:** 1 Department of Vascular Surgery, University Medical Center Utrecht, P.O. Box 85500, 3508 GA Utrecht, The Netherlands; 2 Department of Clinical Chemistry and Haematology, University Medical Center Utrecht, Utrecht, The Netherlands

**Keywords:** Risk factors, Cardiovascular diseases

## Abstract

Extracellular vesicles (EV) are a novel biomarker source for diagnosis and prognosis of cardiovascular disease. A protein comparison of plasma EVs in relation to blood plasma and atherosclerotic plaque has not been performed but would provide insight into the origin and content of biomarker sources and their association with atherosclerotic progression. Using samples of 88 carotid endarterectomy patients in the Athero-Express, 92 proteins (Olink Cardiovascular III panel) were measured in citrate plasma, plasma derived LDL-EVs and atherosclerotic plaque. Proteins were correlated between sources and were related to pre-operative stroke and 3-year major adverse cardiovascular events (MACE). Plasma and EV proteins correlated moderately on average, but with substantial variability. Both showed little correlation with plaque, suggesting that these circulating biomarkers may not originate from the latter. Plaque (n = 17) contained most differentially-expressed proteins in patients with stroke, opposed to EVs (n = 6) and plasma (n = 5). In contrast, EVs contained most differentially-expressed proteins for MACE (n = 21) compared to plasma (n = 9) and plaque (n = 1). EVs appear to provide additional information about severity and progression of systemic atherosclerosis than can be obtained from plasma or atherosclerotic plaque.

## Introduction

Extracellular vesicles (EVs) are a heterogeneous group of bilayer membrane particles that have emerged as a potential novel biomarker source. These secreted encapsulated carriers of biological material act as intercellular messengers of different cell types^1^. Based on their cargo, which reflects the origin cell and comprises of proteins, nucleic acids, lipids and metabolites, EVs have been implicated in cell processes such as inflammation, coagulation, stem cell expansion, neuronal communication and carcinogenesis^2^. As such, the content and composition of EVs can function as a liquid biopsy of cellular processes.

In cardiovascular disease (CVD), EVs have been shown to be associated with traditional risk factors such as smoking, metabolic diseases and hypertension^3–5^. Furthermore, studies demonstrated that EVs can also accumulate in atherosclerotic plaque, where they stimulate foam cell formation, influence smooth muscle cell proliferation and promote endothelial dysfunction, all crucial contributors of atherosclerotic plaque progression^6^. As such, biomarkers in different EV-subpopulations are elevated in patients with myocardial infarction, patients with ischemic stroke, and in cardiovascular patients with future major adverse cardiovascular events (MACE)^7–12^. Albeit a plethora of research on potential pathophysiologic mechanisms and content of EVs is available, a study comparing the protein content of EVs in relation to plasma and atherosclerotic plaque is lacking. These comparisons would elucidate how EV and plasma proteins are expressed in relation to another. A high correlation may consequently reflect a similar (patho)physiological state or origin. In contrast, when the expression of the proteins in these sources is not uniform, the biomarkers may indicate different cellular processes and/or origins. Furthermore, by reviewing the correlation of proteins in plasma and EVs with proteins isolated from atherosclerotic plaque, the extent to which local disease is reflected by circulating biomarkers could be elucidated. This information could guide further biomarker research, and increase our understanding of plasma, EV and plaque content in relation to atherosclerotic disease.

Patients undergoing carotid endarterectomy (CEA) serve as excellent biomaterial donors for this investigation because carotid artery surgery offers the opportunity to procure atherosclerotic plaque tissue as well as plasma and plasma EVs. Although this procedure is performed to reduce the risk of (recurrent) ischaemic cerebral or ocular events in patients with significant carotid stenosis, these patients still have a 13% residual risk of MACE within 3 years. Biomarker analysis at the time of carotid surgery provides both a representation of disease severity at surgery and a clinically relevant starting point for future events in the 3 years after surgery^13^.

Hence, we used citrate plasma and carotid plaque tissue from 88 matched CEA patients that were included in the Athero-Express biobank. For our first objective, we compared protein levels across three biomarker sources: plasma, plasma EVs and atherosclerotic plaque. The secondary objective was to find a differentiation of protein levels in the three biomarker sources between patients with and without stroke and those with and without the 3-year MACE.

## Results

### Cohort selection

**Figure 1:**
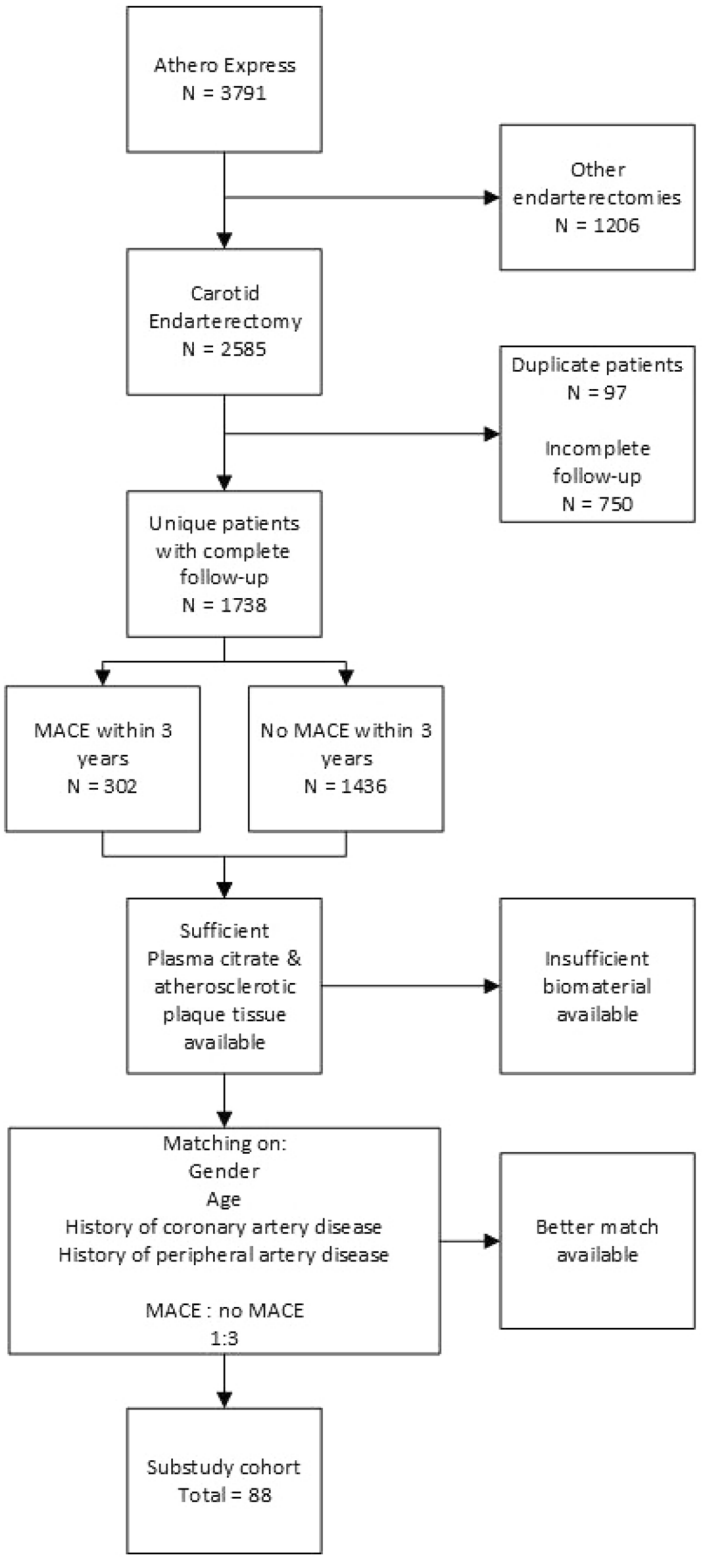
A flow chart of the matched patient’s selection.

Out of the total 3791 patients within the Athero-Express, 88 patients were matched and selected (Fig. 1). Baseline characteristics show that the selected CEA patients represent a normal CEA population with relatively high age (mean age 71 years old), and male predominance (77%) (Table 1). The prevalence of CAD, diabetes mellitus, and history of peripheral interventions is 39.8%, 21.6% and 21.6%, respectively. Adherence to cardiovascular risk management at baseline is high according to the medication prescriptions. In 21 patients, recent stroke was the indication for surgical intervention, opposed to 33 patients with TIA, 15 patients with ocular symptoms and 19 asymptomatic patients. Three years after baseline, 22 patients had a MACE with a median time of 1.47 years (IQR 0.63–2.0).

**Table 1:** Table of baseline characteristics, overall and stratified for MACE.

	Overall	No MACE	MACE	*P*
N	88	66	22	
Age (mean (SD))	71.3 (7.5)	71.3 (7.3)	71.4 (7.9)	.95
Male (%)	68 (77.3)	51 (77.3)	17 (77.3)	1.00
BMI (mean (SD))	26.2 (3.4)	25.8 (3.4)	27.3 (3.2)	.11
Smoking (%)	36 (41.4)	26 (39.4)	10 (47.6)	.68
Pre-operative symptoms				.53
Asymptomatic stenosis	19 (21.6)	15 (22.7)	4 (18.2)	
Ocular	15 (17.0)	9 (13.6)	6 (27.3)	
TIA	33 (37.5)	26 (39.4)	7 (31.8)	
Stroke	21 (23.9)	16 (24.2)	5 (22.7)	
History of				
Peripheral intervention (%)	19 (21.6)	13 (19.7)	6 (27.3)	.65
Coronary artery disease (%)	35 (39.8)	25 (37.9)	10 (45.5)	.71
Stroke (%)	26 (29.5)	20 (30.3)	6 (27.3)	1.00
Hypertension (%)	67 (76.1)	46 (69.7)	21 (95.5)	.030
Diabetes Mellitus (%)	19 (21.6)	12 (18.2)	7 (31.8)	.30
Medication use of				
Insulin (%)	4 (4.5)	2 (3.0)	2 (9.1)	.26
Glucose inhibitors (%)	18 (20.5)	11 (16.7)	7 (31.8)	.22
Anticoagulants (%)	13 (14.8)	10 (15.2)	3 (13.6)	1.00
Antiplatelets (%)	77 (87.5)	58 (87.9)	19 (86.4)	1.00
Lipid lowering drugs (%)	62 (70.5)	48 (72.7)	14 (63.6)	.59
Laboratory results				
eGFR (mean (SD))	70.6 (20.1)	72.8 (21.0)	64.3 (15.7)	.084
Triglycerides (median [IQR])	1.40 [1.04, 1.96]	1.41 [1.02, 1.92]	1.57 [1.09, 2.06]	.46
LDL (mean (SD))	2.35 (0.95)	2.31 (0.889)	2.46 (1.11)	.51
HDL (mean (SD))	1.09 (0.34)	1.11 (0.338)	1.03 (0.35)	.35
Cholesterol (mean (SD))	4.20 (1.16)	4.17 (1.142)	4.27 (1.22)	.72
Plaque features				
Lipid core > 40%	25 (28.4)	18 (27.3)	7 (31.8)	.89
Lipid core > 10%	62 (70.5)	46 (69.7)	16 (72.7)	1.00
Collagen	72 (82.8)	54 (81.8)	18 (85.7)	.94
Smooth muscle cell	58 (66.7)	43 (66.2)	15 (68.2)	1.00
Intraplaque hemorrhage	56 (63.6)	42 (63.6)	14 (63.6)	1.00
Macrophages	49 (57.0)	42 (64.6)	7 (33.3)	.024
MAC mean (mean (SD))	0.855 (1.203)	0.892 (1.161)	0.746 (1.343)	.63
SMC mean (mean ((SD))	2.013 (1.881)	2.084 (2.001)	1.814 (1.516)	.57

*BMI* body mass index, *TIA* transient ischemic attack, *eGFR* estimated glomular filtration rate, *LDL* low-density lipoprotein, *HDL* high-density lipoprotein, *MAC* macrophages count, *SMC* smooth muscle cell, *SD* standard deviation, *MACE* major adverse cardiovascular events.

### Plasma, EV and plaque protein profiles

Of the potential 24.288 protein measurements done with the Olink Cardiovascular III panel in these 88 patients, we identified and removed 385 (0.9%) outliers (> 3SD), with a maximum of 4 outliers per protein.

**Figure 2:**
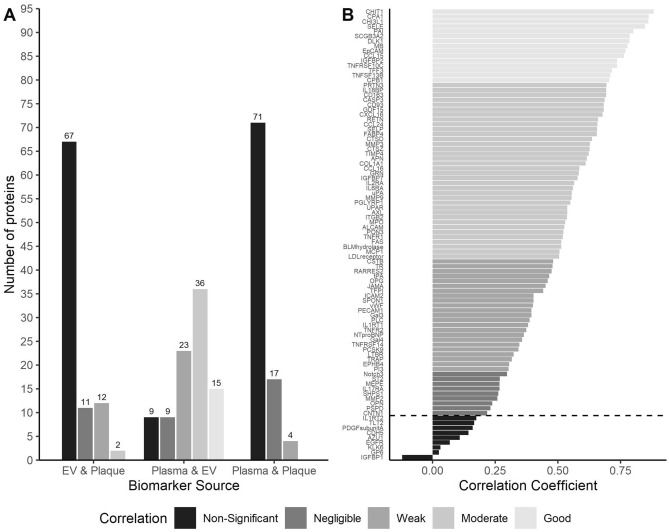
(**A**) Correlations were categorized according to statistical significance and coefficient (negligible, weak, moderate, good and excellent). This was done for all protein correlations between each protein source (EV correlation with plaque, plasma with EV and plasma with plaque). The counts of these categories are given in a bar plot. (**B**) The Spearman’s rho of correlation of plasma and EV are given for all proteins, with their respective categories (negligible, weak, moderate, good and excellent) indicated in greyscale. Proteins under the dashed line are non-significant.

The correlation between plasma and EV proteins was statistically significant in 83 proteins, with a distribution of 9, 23, 36 and 15 proteins showing negligible, weak, moderate and good correlation, respectively (Fig. 2). Correlations of plasma and EV proteins with plaque proteins were hardly significant and often had a much lower r_s_, although significantly more plaque-derived proteins were weakly or moderately (R > 0.30) correlated with EV proteins (15 proteins) than with plasma proteins (4 proteins).

### Differentially-expressed proteins for pre-operative stroke

**Figure 3:**
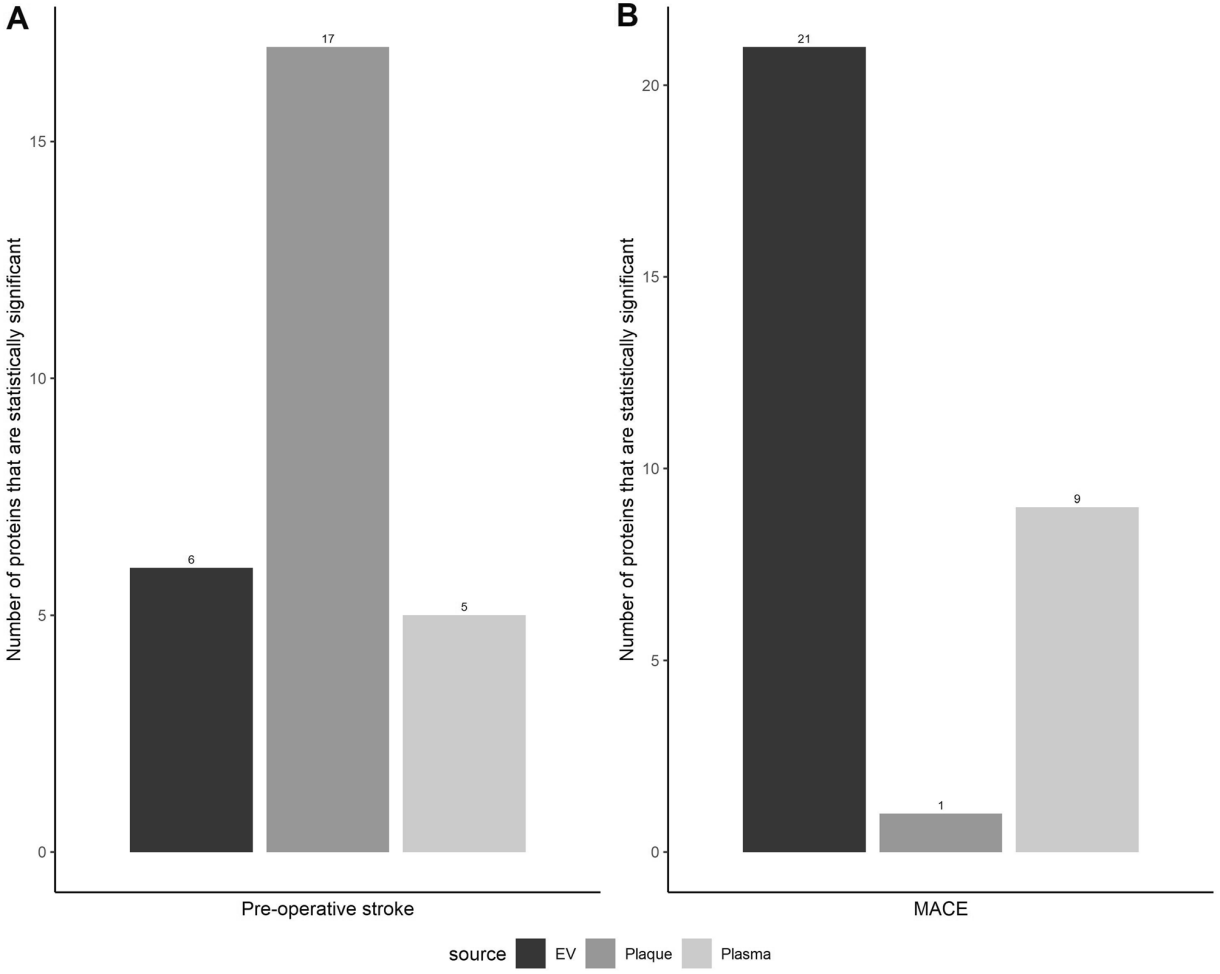
The bars represent the number of differentially-expressed proteins stratified for pre-operative stroke (**A**) and 3-year MACE (**B**). This is done per biomarker source (EV, plaque and plasma), out of the total 94 proteins that were measured.

Protein levels of patients with and without recent pre-operative stroke, were compared in plasma, EV and plaque, in order to identify differentially-expressed proteins (DEPs) (Supplemental TABLES 1A-B). Plaque (N = 17) contained the most DEPs opposed to the plasma (N = 5) and EVs (N = 6) (Fig. 3). The levels of DEPs in plaque and EV were higher (except for PAI) in patients with pre-operative stroke. In contrast, plasma fraction protein levels were lower in patients with pre-operative stroke compared to patients without pre-operative stroke.

### Differentially-expressed proteins for MACE

**Figure 4:**
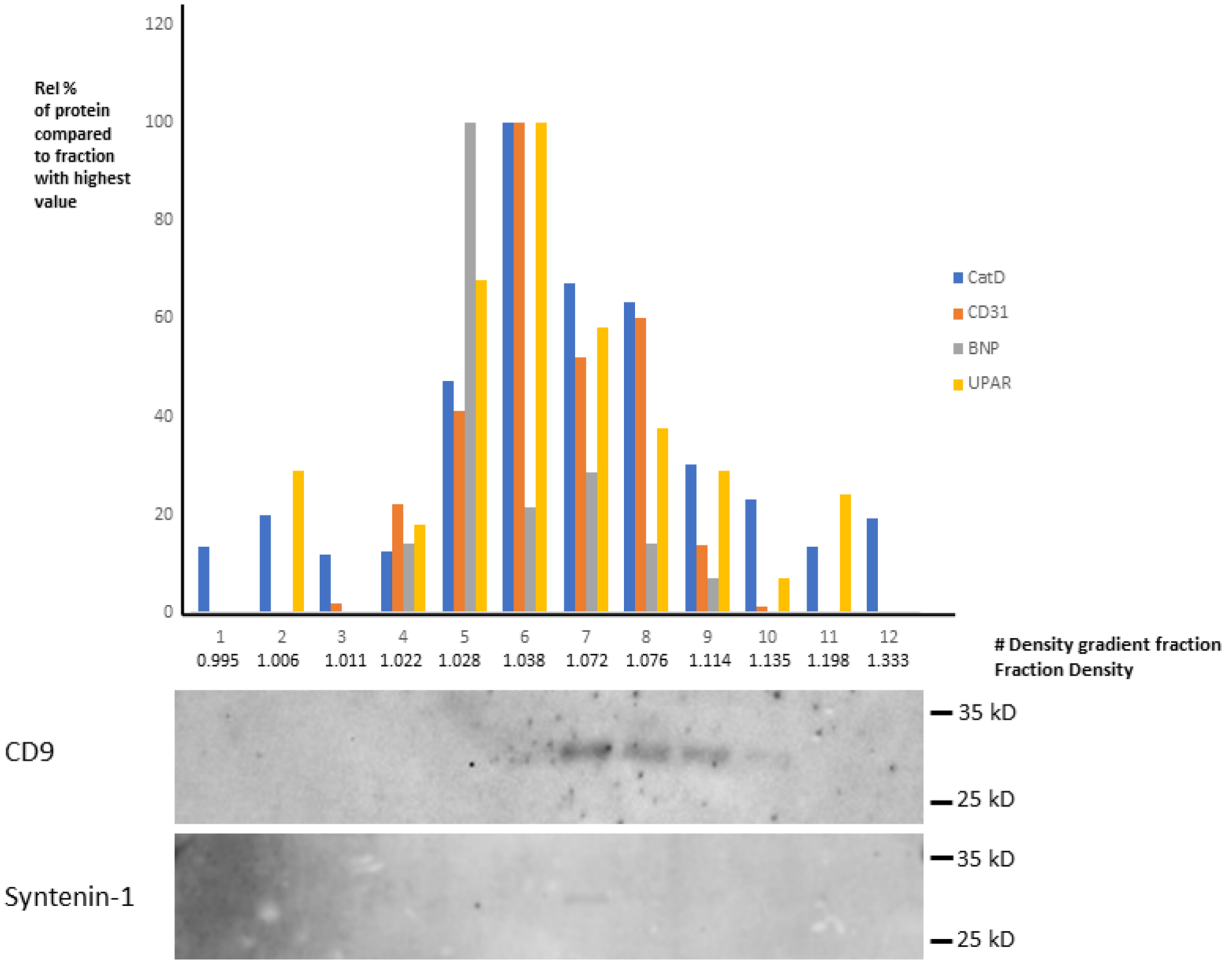
The bar plot at the top shows density gradients for 12 fractions of CTSD, CD31, BNP and UPAR. Below is the western blot analysis in which CD9 vesicle markers are present in fractions 7–10.

With regards to 3-year MACE following surgery, the EV-fraction (N = 21) had more DEPs compared to the plasma (N = 9) and plaque fractions (N = 1) (Fig. 4; Supplementary tables 2A-B). Fifteen proteins were differentially-expressed in the EV-fraction only, whereas six DEPs were found in both the EV and plasma fractions. Of these six corresponding proteins, correlation between EV and plasma was weak (N = 2), moderate (N = 2) and good (N = 2). In all sources, these DEPs were higher in patients with MACE compared to patients without.

### Association of plaque proteins with plaque characteristics

The seventeen DEPs for pre-operative stroke were compared with plaque characteristics. Ten out of these 17 proteins were higher in plaque with a lipid core, compared to plaque without a lipid core. Four proteins were higher when stratified for the presence of smooth muscle cells (IL18BP, GDF15, TR, MCP1), one was higher in collagen-rich plaque (MCP1) and plaque with more macrophages (CASP3). The single DEP from plaque, that was elevated in patients with MACE (PON3), was not associated with semi quantitative plaque features.

## Discussion

This study shows that, within a small matched cohort of patients undergoing CEA, the majority of Olink cardiovascular panel III proteins measured in plasma and EVs show moderate to good correlations, although the range of correlations is wide. Proteins from plaque have little correlation with either EV or plasma fraction, but are most frequently differentially-expressed when stratified for pre-operative-stroke. In contrast, stratifying protein levels for MACE within 3 years after surgery, EVs contain the most differentially-expressed proteins.

It has already been mentioned that our understanding of biomarkers in different sources is extremely limited. Our data would suggest that there are in fact both similarities and dissimilarities of (cardiovascular) protein markers in plasma compared to EVs. Consequently, the expression of well-correlated proteins may be the result of equal cellular processes, or a resemblance of cell-specific origin. The latter was to be expected in some plasma and EV proteins, as EVs in this study were isolated from plasma. On the other hand, some of these proteins might reflect different pathophysiological processes, or different origins and/or targets, when comparing plasma to EVs as sources. The lower degree of correlation between some plasma and EV proteins and their dissimilar association with MACE could indicate that the cell-specific origin of some EV proteins may be different from that of their equivalent plasma proteins. This has not been evaluated in studies, and should be considered an objective for future research^14^. In effect, when a protein is identified as a potential biomarker, multiple sources should be explored to rule out its discriminatory effect.

An equally wide range of correlations between EVs and plasma has been reported in another study that used the Olink platform, when they compared protein expression in patients (N = 82) with myocardial infarction to controls^11^. Tumor necrosis factor ligand superfamily member 13B (TNFSF13B) was also measured in that study, and showed a similar correlation coefficient (0.71 according to our data, 0.75–0.8 according to theirs), underlining that the correlations we observed are not coincidental. Unfortunately, that protein was the only overlapping marker with our study so general conclusions cannot be deduced from these data. Other studies that looked at specific EV biomarkers, rather than a large selection of biomarkers, likewise showed that plasma-derived and EV-derived proteins show varying correlations^15–17^. However, these specific proteins were not used in our analyses and thus the correlations cannot be compared with our data.

As for plaque, the correlation with either plasma or EV is limited, rendering it a more unique source. In our analyses, EV proteins correlated better with plaque proteins than plasma proteins. Since EVs have also been proven to emanate from atherosclerotic plaque, it could be hypothesized that EV proteins originate from plaque in a greater extent compared to plasma, and consequently reflect its pathophysiological state better^4,18^. Taking an EV liquid biopsy of systemic plaque proteins can therefore provide an accurate impression of the plaque content.

However, since atherosclerotic plaques are not easily available as a regular biomarker source, a direct comparison of blood biomarkers with plaque has rarely been performed and thus this remains ambiguous. Equivalent research is restricted to one study (N = 574), published by our research group, which determined the correlation coefficient of osteopontin (OPN) in plasma and plaque (r_s_ = 0.15). Coincidentally, OPN was also measured with the current Olink CVD panel III, and the correlation between sources appears to be almost identical (r_s_ = 0.16)^19^.

Our secondary objective was to determine whether a disparity in protein content across these sources would result in a difference in discriminatory power for a diagnostic criterion (pre-operative stroke) and future outcomes (MACE within 3 years following surgery). Concerning pre-operative stroke, our results indicate that plasma and EVs contained a limited number of DEP. Statistically significant proteins in EVs were higher in stroke patients, but conversely, proteins in plasma were significantly lower in the affected group, compared to the patients without stroke. Our study design is not suitable to elucidate this inverse relationship specifically, however, it has described that Kallikrein Related Peptidase 6 (KLK6) and Plasminogen activator inhibitor (PAI) are decreased during subarachnoid bleeding and 1-week after stroke, respectively^20,21^. Furthermore, our results are perhaps influenced by the timing of blood sampling, as this did not take place during the stroke but in the subsequent period after initial diagnosis and treatment.

A significantly larger number of plaque proteins are differentially-expressed in patients with pre-operative stroke, compared to plasma and EV. Since the harvested atherosclerotic plaque is often considered the culprit lesion for cerebrovascular events, this comes as little surprise^22^. Although this has not been tested in other research, higher plaque proteins levels are related to characteristics of plaque instability in several studies, which are in turn associated with stroke^23–26^. In addition, we found that most of these differentially-expressed plaque proteins are also statistically higher in plaques with a prominent lipid profile, underlining that plaque histology is reflected by protein expression.

With regards to the discriminatory power of future events, only one plaque-derived protein was differentially-expressed for 3-year MACE. This might indicate that the ongoing systemic processes associated with these events are not properly represented by the disease state of the carotid plaque alone, but might be captured more accurately with systemic (EV) biomarkers. This is demonstrated with our data, as there are twenty-one differentially-expressed EV-proteins, compared to nine differentially-expressed proteins for plasma. Again, no comparative analysis of multiple biomarker sources has been performed, let alone demonstrated the predictive properties of these biomarkers with respect to any long-term outcome.

### Strengths and limitations

The Athero-Express biobank, with well-defined baseline descriptions and the possibility of using both blood and atherosclerotic plaque, makes it feasible to create a matched cohort of 88 patients and enough biomaterial for multiple analyses. Obviously, a larger selection of patients would have been preferred, although equivalent studies used less patients^11^. Furthermore, the insight we provide is limited to only 92 selected cardiovascular selected proteins, which amounts to only a fraction of the entire EV, plasma and plaque proteomes. The EV precipitation method is an enrichment for EV proteins and as such, it yields not only pure EV proteins, but also proteins in EVs, on EV membranes, in EV corona and plasma^27^. For this EV isolation, the LDL fraction was preferred, as it is our experience that this fraction produces the most protein. As such, EVs in other fractions might show different correlations with plasma or plaque, or different expression when stratified for pre-operative stroke and outcome.

Olink is optimized for plasma and thus measurements for EV and plaque proteins might lead to more measurements below the limit of detection (LoD), employed by Olink. While these LoDs seem very specific and critical, their recommended utility is more nuanced, as the manufacturer suggests that proteins should be considered for exclusion when less than 25–50% of the proteins are above the LoD. Furthermore, as stated by Olink, the inclusion of data under LoD does not commonly increase false positives^28^. We have addressed this in the supplementary files and thus demonstrate that analysis of the data above the LoD does not change our general conclusions. Furthermore, although measurements of Cathepsin D are fully below the LoD it correlates well with MSD immunoassay measurements of the same samples, substantiating our hypothesis that the full data can in fact be used.

Applying these strict boundaries would lead to less viable measurements of EV proteins and plaque proteins especially. However, in the supplemental material a brief overview of these remaining data shows that our general conclusions still stand.

Since plasma samples are centrifugated only once at low speed this might lead to platelet contaminations. This potential platelet contamination, however, is relatively equal in plasma, EV isolations and plaque extracts and hence this could mask the association of protein level with MACE and presurgical stroke in all three protein sources.

Regarding the analyses of pre-operative stroke and 3-year MACE, our protein-specific results should be interpreted with caution. Selection bias (a matched cohort) and limited power impair direct conclusions about the efficacy of these potential biomarkers. Moreover, the objective of this article is to examine how cardiovascular proteins from different protein sources in one individual are associated with MACE and pre-surgical stroke. Although potential proteins will indeed yield predictive performance in this matched cohort, these analyses should be performed in a large cohort of patients, to obtain enough patient numbers and thus statistical power, and to enable multivariable regression analysis and multiple testing correction.

## Conclusion

Although the correlation between plasma and EV proteins is moderate overall, there are protein-specific differences, which may reflect that both sources yield proteins from different cellular processes or origins. In contrast, very little correlation of either circulating source was seen with plaque, although the atherosclerotic plaque, often seen as the culprit for stroke, contained more proteins differentially-expressed for stroke. EVs contain the most DEPs for 3-year MACE, indicating that EVs are indeed a relatively unexplored source of systemic protein biomarkers that, compared to plasma, provide additional information on cardiovascular disease severity.

## Methods

### Study population and design

The Athero-Express Biobank Study (AE) is an ongoing prospective vascular biobank with a collection of biological materials such as blood and atherosclerotic plaque, in addition to baseline characteristics and 3-year follow-up data. Patients undergoing CEA or femoral endarterectomy in two hospitals located in the Netherlands (UMC Utrecht and St. Antonius Hospital, Nieuwegein) are eligible for inclusion in the AE. The study design has been described in more detail^29^. The study has been approved by the Institutional Review boards of both hospitals and written informed consent was obtained from all patients. The study is conducted in accordance with the declaration of Helsinki^30^.

All patients who were included in the AE and underwent CEA, with complete 3-year follow-up, were eligible for inclusion in this sub study. Exclusion criteria were lack of citrate plasma, plaque, or follow-up data. Patients were matched for the presence of MACE and no MACE, based on gender, age, history of coronary artery disease and the presence of peripheral artery disease. A MACE/no MACE ratio of 1:3 was chosen because it would facilitate statistical testing with a smaller sample size, even though it is slightly higher than the overall prevalence of MACE in the Athero-Express (13%).

### Blood collection, tissue collection and plaque processing

Venous blood was collected in citrate tubes the day before surgery. Citrate tubes were centrifuged (10 min, 1850×g @ room temperature (RT)) within 30 min after collection. Plasma was aliquoted and directly stored at − 80 °C.

Freshly dissected carotid plaques were divided into 0.5 cm segments and processed following standard procedure^29^. Plaques were grinded in liquid nitrogen and approximately 500 μl of residue was used for the extraction of proteins with 500 μl 40 mM Tris buffer (pH 7.5) and an 1 × EDTA-free proteinase inhibitor cocktail (Roche) and 20 s of iced sonification.

After centrifugation (10 min, 13 krpm @ 4 °C), the supernatant was stored at − 80 °C, as the Tris fraction.

#### Isolation of extracellular vesicle plasma subfractions

LDL subfraction was obtained from citrate plasma using Dextrane Sulphate (DS) of 0.05% (end concentration) and MnCl_2_ of 0.05 M (end-concentration). For this, DS and MnCl_2_ were added to phosphate buffered saline (PBS) (Gibco) giving a volume of 95 µL. Next 5 µL magnetic beads (Nanomag®-D plain, 130 mm (1:25) (Micromod)) were added followed by the addition of 25 µL citrate plasma and mixed. The mixture was incubated 5 min at room temperature (RT). Subsequently, the samples were placed on a bio-plex handheld magnet (Bio-Rad) and incubated 15 min at RT. After removal of the supernatant, pellets were lysed with 125 µL Roche complete lysis-M with protease inhibitors (Roche). To remove magnetic beads and other debris, samples were centrifuged (10 min, 3200×g @ RT).

### Characterization of plasma extracellular vesicles subfractions

EV characterization in the plasma EV subfractions has been reported previously^31–33^. For this study, we performed additional experiments in order to confirm the presence of proteins (Urokinase receptor (UPAR), NT-proBNP, CD31 and Cathepsin D (CTSD)) in EVs with an electrochemiluminescence immunoassay (Quickplex SQ120, Meso Scale). Density gradient centrifugation of the LDL EV subfraction resulted in 12 density gradient fractions.

CD9 (Santa Cruz Biotechnology #SC13118, primary antibody) and Syntenin-1 (Novusbio, #nb100-53,807 as primary antibody) western blot analysis showed that vesicles markers are present in fractions 7–10 with densities between 1.07 and 1.13. Our Mesoscale measurements of UPAR, NT-proBNP, CD31 and CTSD proteins show that these protein concentrations are primarily in density gradient fractions 5–10 which largely overlaps with the EV markers (Fig. 4).

### Blood collection and protein measurements

The multiplex OLINK® proteomics immunoassays Cardiovascular III panel (OLINK® Proteomics, Uppsala, Sweden) was used to simultaneously determine 92 cardiovascular disease-related biomarker proteins. A proprietary Proximity Extension Assay (PEA) technology was used to achieve high-level multiplexing, which transforms the protein values into Normalised Protein eXpression (NPX), a relative unit on a log2 scale. These NPX cannot be converted into absolute protein concentrations. All information regarding detection limits, assay performance and validation are available on the manufacturer’s website (www.olink.com). Olink measurements from each protein source were done on a separate Olink plate.

For this analysis, we excluded all protein values that exceeded 3 standard deviations (SD). Our primary analyses have included proteins, regardless of their LoD. This is further covered in the strengths and limitations section of the discussion.

### Follow-up and clinical outcome

After initial CEA, patients included in the AE underwent 3-year follow-up, through annual questionnaires. When patients did not respond to the questionnaire, their general practitioner was contacted. Verification of the outcome event was performed by an outcome event committee. The primary endpoint was MACE, a composite of nonfatal myocardial infarction and stroke, and cardiovascular death. Cardiovascular death was defined as one of the following: fatal myocardial infarction, fatal stroke (either haemorrhagic or ischemic), fatal ruptured abdominal aneurysm, fatal heart failure or sudden death. Only the first manifestation of a cardiovascular event was used for analysis.

### Statistical analysis

Descriptive statistics of baseline characteristics between groups were compared using t-test, Mann–Whitney U test, Chi-squared test or Fisher’s exact test according to variable type and their respective distribution.

Correlations of proteins between the different biomarker sources were analysed by calculating Spearman’s rho (r_s_) correlation coefficients. These were interpreted as follows: 0.0–0.29 negligible, 0.3–0.49 weak, 0.50–0.69 moderate, 0.7–0.9 good, and > 0.9 excellent correlation.

For the secondary objective, a Cox proportional hazard (PH) regression model was used for endpoint analysis. Multiple univariable regression was performed to find potential confounders.

All data were analysed with R (R Core Team (2017) version 3.6.2, Vienna, Austria). A two-sided *P*-value of < 0.05 was considered significant. No multiple testing correction has been performed.

## Supplementary Information

Supplementary Information 1.

Supplementary Figure S1.

Supplementary Figure S2.
