# Supplementary material for: Comparison of cardiovascular biomarker expression in extracellular vesicles, plasma and carotid plaque for the prediction of MACE in CEA patients
Source: Sci Rep. 2023 Jan 18;13:1010. doi: 10.1038/s41598-023-27916-6 (PMC9849473; doi:10.1038/s41598-023-27916-6)
Supplement: Supplementary file 1 — Supplementary Information 1. [file 41598_2023_27916_MOESM1_ESM.docx]

Contents of supplementary material

[Availability of Data and Materials 1](#_Toc124066345)

[Supplementary text: 1](#_Toc124066346)

[Supplementary images: 2](#_Toc124066347)

[Supplementary Tables 1A-B: 3](#_Toc124066348)

[Supplementary Tables 2A-B: 6](#_Toc124066349)

### Availability of Data and Materials

The datasets generated and/or analysed during the current study are available in the DataverseNL repository upon request, [<https://doi.org/10.34894/W8YBH6>](https://doi.org/10.34894/W8YBH6).

### Supplementary text:

**Considering the limit of detection (LoD) in relation to our measurements**

For our main paper, the LoD was ignored as these values were based on plasma-matrix. Furthermore, Olink indicated that exclusion could be considered, but is not mandatory, when there are less than 25-50% proteins above the LoD. Olink proposes several ways to deal with results below the LoD. These include using the actual data below LoD, replace these values with a pre-determined value and impute these data. In our main paper we opted for the first recommendation, to ensure the robustness of our results, we checked what impact LoD would have on our data.

As such, we excluded all measurements below the LoD and only included proteins that had more than 75% (N = 66) measurements above the LoD, in order to assure some statistical power.

|  | Fully omitted  proteins (N) | Proteins with all measurements  > LoD (N) | Included proteins (N) |
| --- | --- | --- | --- |
| Plasma | 23 | 61 | 69 |
| EV | 63 | 22 | 29 |
| Plaque | 55 | 25 | 37 |

As can be expected, the removal of proteins below the LoD had the least influence on plasma as these absolute values were highest compared to EV and plaque.

For plasma, extracellular vesicles (EVs) and plaque 69, 29 and 37 proteins could be analysed, respectively. These data were used to statistically test the significant association with regards to major adverse cardiovascular events (MACE) and pre-operative stroke, which was part of our manuscript.

For pre-operative stroke the number of EV-proteins was downgraded (6 out of 92 (6%) in the main analyses, *vs.* 0 out of 29 with (0%) >75% above LoD). The relative number of statistically significant proteins remained unchanged for plasma (5 out of 92 (2%) in the main analyses, *vs.* 2 out of 69 (3%) with >75% above LoD) and plaque (17 out of 92 (18%) in the main analyses, *vs.* 8 out of 37 (21%) with >75% above LoD). This again shows (as can be expected since the carotid plaque is often the underlying cause of stroke) that more plaque proteins are associated with symptomatic stroke compared to the same proteins in plasma and EVs.

The relative number of statistically significant EV proteins for MACE remains similar (21 out of 92 (23%) in the main analyses, *vs.* 7 out of 29 (24%) with >75% above LoD). For plasma proteins, this relative number was raised marginally (9 out of 92 (10%) in the main analyses, *vs.* 11 out of 69 (15%) with >75% above LoD). Plaque had a limited number of statistically significant proteins for MACE in our main analyses and this was not different when taking the LoD into consideration (1 out of 92 (1%) in the main analyses, *vs.* 0 out of 37 (0%) with >75% above LoD). This again shows that more EV proteins are associated with MACE then plasma and plaque.

**Considering the LoD and cross-platform validity**

The mesoscale discovery (MSD) was used to compare Cathepsin D (CTSD) with the Olink CTSD data, as all 88 samples were below the LoD in both EV and plasma. This cross-platform analysis shows a significant correlation coefficient of 0.602 and 0.705 for EV and plasma respectively, which substantiates the hypothesis that the measurements below LoD are valid.

### Supplementary images:

Attached to the supplementary files are the two full-length Western Blots of CD9 (Santa Cruz Biotechnology #SC13118, primary antibody) and Syntenin-1 (Novusbio, #nb100-53807 as primary antibody) that are cropped in the main manuscript (figure 4).

### Supplementary Tables 1A-B:

For the following 2 tables, EV, plasma and plaque proteins are stratified for pre-operative stroke. These proteins are measured with the Olink Cardiovascular III panel. Non-parametric Mann-Whitney U test is performed with protein expression given as median [interquartile range].

For the abbreviations of proteins, we refer to Olink’s website: <https://www.olink.com/content/uploads/2021/09/1023-v1.3-cvd-iii-panel-content-final.pdf>

|  | *EV* | | | *Plasma* | | |
| --- | --- | --- | --- | --- | --- | --- |
|  |  | | |  | | |
|  | *No Stroke* | *Stroke* | *P* | *No Stroke* | *Stroke* | *P* |
|  | N = 67 | N =21 |  | N = 67 | N = 21 |  |
| TNFRSF14 | 1.61 [1.34, 1.86] | 1.77 [1.48, 1.90] | .13 | 17.90 [15.42, 22.71] | 18.81 [15.12, 24.76] | .86 |
| LDLreceptor | 0.41 [0.35, 0.51] | 0.43 [0.39, 0.49] | .57 | 8.44 [6.02, 11.20] | 7.50 [5.70, 8.89] | .28 |
| ITGB2 | 0.99 [0.79, 1.14] | 1.01 [0.87, 1.12] | .66 | 15.63 [12.43, 20.23] | 13.88 [12.57, 15.57] | .25 |
| IL17RA | 0.97 [0.77, 1.09] | 1.04 [0.91, 1.11] | .20 | 8.12 [6.52, 11.10] | 8.84 [6.04, 10.64] | .87 |
| TNFR2 | 2.40 [1.87, 2.76] | 2.31 [1.99, 3.05] | .55 | 26.28 [21.97, 37.02] | 27.22 [21.68, 46.02] | .34 |
| MMP9 | 0.70 [0.62, 0.84] | 0.82 [0.65, 0.88] | .30 | 10.68 [6.43, 13.90] | 10.20 [7.62, 17.29] | .82 |
| EPHB4 | 1.85 [1.60, 2.02] | 1.85 [1.44, 2.32] | .98 | 26.61 [22.77, 31.60] | 27.22 [21.04, 35.74] | .72 |
| IL2RA | 0.70 [0.63, 0.80] | 0.76 [0.67, 0.85] | .39 | 10.16 [7.60, 12.96] | 10.29 [7.12, 12.52] | .94 |
| OPG | 0.80 [0.69, 0.95] | 0.83 [0.72, 1.00] | .51 | 12.26 [9.39, 14.11] | 13.06 [9.85, 15.95] | .51 |
| ALCAM | 3.28 [2.73, 3.68] | 3.15 [2.40, 3.35] | .27 | 110.26 [89.75, 129.82] | 105.76 [85.85, 127.13] | .87 |
| TFF3 | 1.45 [1.19, 1.70] | 1.45 [1.13, 1.59] | .59 | 24.99 [19.24, 30.98] | 23.43 [16.72, 27.22] | .55 |
| SELP | 13.91 [10.31, 18.53] | 11.94 [10.39, 17.83] | .58 | 556.18 [465.84, 721.11] | 533.12 [332.10, 769.62] | .70 |
| CSTB | 0.54 [0.48, 0.64] | 0.51 [0.45, 0.60] | .29 | 9.82 [7.56, 13.54] | 9.54 [6.73, 13.49] | .91 |
| MCP1 | 0.97 [0.75, 1.12] | 1.02 [0.88, 1.18] | .15 | 14.01 [10.36, 16.87] | 14.41 [10.62, 19.52] | .84 |
| CD163 | 3.64 [2.94, 4.70] | 4.00 [3.20, 4.38] | .85 | 113.65 [97.93, 151.52] | 120.27 [98.36, 146.18] | .97 |
| Gal3 | 0.23 [0.20, 0.26] | 0.22 [0.19, 0.23] | .33 | 6.92 [5.62, 8.64] | 6.58 [6.02, 8.94] | .78 |
| GRN | 1.29 [1.18, 1.43] | 1.31 [1.15, 1.40] | .92 | 29.86 [24.32, 33.52] | 27.99 [20.75, 33.01] | .60 |
| NTproBNP | 2.17 [1.78, 2.44] | 2.50 [2.05, 2.75] | .018 | 13.39 [6.58, 21.75] | 14.11 [9.38, 46.00] | .36 |
| BLMhydrolase | 0.20 [0.18, 0.22] | 0.19 [0.16, 0.24] | .39 | 2.84 [2.41, 3.40] | 2.80 [2.33, 3.24] | .38 |
| PLC | 4.26 [3.64, 6.37] | 4.42 [3.70, 5.08] | .90 | 218.23 [181.74, 281.26] | 187.01 [155.02, 290.23] | .31 |
| LTBR | 0.57 [0.46, 0.67] | 0.59 [0.50, 0.63] | .62 | 7.79 [6.30, 9.46] | 8.15 [6.16, 11.03] | .83 |
| Notch3 | 1.06 [0.92, 1.20] | 1.02 [0.92, 1.26] | .88 | 22.32 [16.28, 30.03] | 20.00 [17.87, 29.41] | .68 |
| TIMP4 | 0.53 [0.46, 0.63] | 0.63 [0.51, 0.74] | .015 | 9.74 [7.43, 11.69] | 9.71 [8.22, 13.10] | .48 |
| CNTN1 | 0.40 [0.35, 0.47] | 0.39 [0.35, 0.43] | .45 | 9.27 [7.63, 11.40] | 9.64 [7.29, 11.22] | .78 |
| CDH5 | 1.46 [1.22, 1.68] | 1.49 [1.33, 1.70] | .67 | 11.88 [9.56, 14.51] | 12.26 [8.06, 15.90] | .97 |
| TLT2 | 2.06 [1.64, 2.56] | 2.27 [1.76, 2.69] | .26 | 18.24 [14.29, 22.49] | 16.69 [12.87, 21.02] | .39 |
| FABP4 | 1.14 [1.03, 1.58] | 1.12 [0.91, 1.56] | .57 | 36.56 [27.14, 50.30] | 32.86 [25.95, 60.92] | .99 |
| TFPI | 10.03 [7.66, 11.27] | 9.37 [7.58, 11.85] | .95 | 388.41 [294.84, 467.99] | 324.74 [263.78, 404.16] | .075 |
| PAI | 0.64 [0.54, 0.78] | 0.56 [0.48, 0.62] | .017 | 15.97 [11.63, 26.24] | 11.42 [8.79, 16.20] | .047 |
| CCL24 | 0.68 [0.50, 0.82] | 0.76 [0.68, 0.81] | .058 | 19.97 [13.19, 32.18] | 20.98 [15.95, 33.39] | .41 |
| TR | 1.42 [1.24, 1.71] | 1.51 [1.10, 1.75] | .99 | 14.48 [10.87, 20.02] | 14.33 [11.11, 21.60] | .98 |
| TNFRSF10C | 3.23 [2.62, 4.19] | 2.89 [2.45, 3.98] | .56 | 57.13 [44.75, 70.14] | 59.07 [40.46, 72.30] | .86 |
| GDF15 | 1.20 [0.99, 1.51] | 1.35 [1.00, 1.92] | .31 | 49.64 [37.70, 68.02] | 51.84 [33.29, 85.35] | .78 |
| SELE | 79.01 [57.79, 105.80] | 66.43 [48.50, 84.85] | .22 | 2397.13 [1847.53, 3343.31] | 2372.53 [1654.03, 2733.01] | .29 |
| AZU1 | 0.28 [0.24, 0.31] | 0.29 [0.23, 0.35] | .44 | 4.65 [3.27, 8.04] | 3.99 [2.96, 7.15] | .33 |
| DLK1 | 2.00 [1.62, 2.51] | 1.98 [1.52, 2.45] | .59 | 46.38 [29.84, 61.37] | 35.74 [26.25, 46.85] | .23 |
| SPON1 | 1.51 [1.30, 1.74] | 1.65 [1.39, 1.78] | .23 | 2.35 [2.04, 2.81] | 2.19 [1.92, 2.51] | .13 |
| MPO | 0.59 [0.53, 0.65] | 0.65 [0.56, 0.69] | .10 | 7.30 [5.32, 9.31] | 7.93 [5.52, 9.80] | .77 |
| CXCL16 | 1.52 [1.25, 1.85] | 1.65 [1.38, 1.99] | .38 | 28.01 [22.09, 35.56] | 33.51 [19.42, 35.45] | .80 |
| IL6RA | 49.27 [39.50, 55.65] | 45.65 [30.41, 65.49] | .45 | 3060.01 [2353.02, 3954.50] | 2963.45 [2257.26, 3380.08] | .44 |
| RETN | 0.98 [0.81, 1.31] | 1.12 [0.85, 1.44] | .53 | 64.45 [46.89, 78.82] | 64.83 [43.97, 80.57] | .96 |
| IGFBP1 | 1.13 [0.91, 1.30] | 1.21 [0.95, 1.56] | .23 | 17.54 [10.27, 31.26] | 17.36 [10.22, 61.08] | .75 |
| CHIT1 | 1.39 [1.16, 1.74] | 1.56 [1.12, 1.80] | .73 | 31.39 [19.44, 39.72] | 31.68 [19.72, 43.18] | .60 |
| TRAP | 0.32 [0.28, 0.37] | 0.33 [0.29, 0.37] | .50 | 7.50 [5.68, 8.93] | 7.63 [5.81, 9.21] | .71 |
| GP6 | 0.44 [0.40, 0.53] | 0.48 [0.41, 0.54] | .28 | 1.70 [1.43, 2.24] | 1.60 [1.29, 1.89] | .24 |
| PSPD | 0.59 [0.49, 0.71] | 0.60 [0.47, 0.71] | .88 | 4.29 [2.53, 6.66] | 2.78 [1.99, 3.89] | .023 |
| PI3 | 0.33 [0.30, 0.39] | 0.34 [0.29, 0.35] | .39 | 2.75 [2.10, 3.96] | 2.19 [1.86, 3.18] | .14 |
| EpCAM | 1.26 [0.99, 1.73] | 1.16 [0.91, 1.60] | .27 | 24.64 [14.01, 48.78] | 20.84 [10.91, 26.90] | .12 |
| APN | 1.05 [0.91, 1.18] | 0.97 [0.82, 1.23] | .46 | 20.92 [16.27, 26.53] | 20.99 [17.08, 23.91] | .61 |
| AXL | 7.61 [6.33, 8.58] | 8.07 [6.26, 9.09] | .83 | 213.03 [177.89, 269.13] | 209.03 [184.25, 235.79] | .59 |
| IL1RT1 | 1.77 [1.51, 2.09] | 1.81 [1.42, 1.90] | .51 | 46.55 [38.52, 54.57] | 45.41 [37.02, 52.80] | .68 |
| MMP2 | 0.61 [0.55, 0.65] | 0.60 [0.57, 0.67] | .47 | 7.24 [5.21, 8.52] | 6.21 [5.64, 7.90] | .48 |
| FAS | 0.86 [0.73, 0.98] | 0.87 [0.75, 1.02] | .64 | 30.03 [23.68, 34.97] | 25.30 [21.86, 36.42] | .33 |
| MB | 3.11 [2.59, 4.70] | 3.42 [2.48, 3.96] | .97 | 113.23 [80.85, 163.44] | 104.42 [77.61, 163.73] | .79 |
| TNFSF13B | 3.08 [2.57, 3.78] | 2.76 [2.36, 3.82] | .20 | 92.46 [72.30, 126.05] | 79.41 [59.70, 93.64] | .094 |
| PRTN3 | 0.42 [0.36, 0.51] | 0.53 [0.42, 0.59] | .009 | 8.00 [5.65, 10.97] | 8.42 [6.78, 16.51] | .058 |
| PCSK9 | 0.82 [0.71, 0.99] | 0.88 [0.75, 0.98] | .26 | 4.59 [3.93, 5.91] | 4.72 [3.58, 5.90] | .74 |
| UPAR | 1.72 [1.48, 2.01] | 1.70 [1.51, 1.93] | .80 | 29.26 [21.33, 37.00] | 27.67 [22.80, 35.86] | .89 |
| OPN | 0.71 [0.62, 0.83] | 0.81 [0.68, 0.94] | .080 | 97.71 [74.65, 137.10] | 90.72 [80.88, 162.00] | .67 |
| CTSD | 0.25 [0.22, 0.28] | 0.25 [0.22, 0.32] | .74 | 5.61 [4.08, 6.64] | 5.55 [3.78, 6.77] | .85 |
| PGLYRP1 | 2.14 [1.61, 2.51] | 2.29 [2.02, 2.67] | .19 | 116.53 [86.64, 152.67] | 104.58 [85.65, 153.64] | .57 |
| CPA1 | 1.06 [0.81, 1.43] | 0.94 [0.81, 1.17] | .22 | 36.47 [24.73, 50.22] | 28.46 [17.19, 40.45] | .087 |
| JAMA | 0.46 [0.38, 0.61] | 0.45 [0.32, 0.53] | .11 | 11.19 [9.10, 14.00] | 8.35 [7.44, 13.25] | .051 |
| Gal4 | 0.82 [0.72, 1.01] | 0.85 [0.80, 0.96] | .72 | 9.44 [7.57, 14.60] | 8.89 [6.66, 10.95] | .27 |
| IL1RT2 | 1.01 [0.85, 1.25] | 0.93 [0.88, 1.12] | .34 | 23.28 [19.32, 26.73] | 20.94 [18.39, 27.30] | .58 |
| SHPS1 | 0.86 [0.78, 0.98] | 0.86 [0.80, 0.97] | .85 | 7.48 [5.63, 9.04] | 6.55 [6.07, 8.21] | .27 |
| CCL15 | 2.01 [1.58, 2.46] | 1.96 [1.67, 2.21] | .88 | 112.02 [86.78, 166.77] | 109.79 [79.92, 141.05] | .32 |
| CASP3 | 0.95 [0.70, 1.36] | 0.88 [0.69, 1.23] | .63 | 34.38 [20.34, 56.22] | 23.54 [14.59, 30.73] | .046 |
| uPA | 0.62 [0.51, 0.71] | 0.65 [0.55, 0.74] | .60 | 16.94 [13.11, 20.91] | 15.54 [11.30, 19.29] | .34 |
| CPB1 | 0.88 [0.74, 1.01] | 0.83 [0.71, 0.98] | .43 | 29.72 [24.29, 45.96] | 22.81 [15.58, 36.05] | .11 |
| CHI3L1 | 0.31 [0.22, 0.46] | 0.40 [0.29, 0.84] | .045 | 12.68 [6.76, 19.65] | 12.49 [8.29, 25.35] | .32 |
| ST2 | 1.61 [1.22, 1.91] | 1.86 [1.31, 2.27] | .15 | 13.40 [10.99, 16.70] | 16.35 [10.23, 20.51] | .49 |
| tPA | 1.53 [1.25, 1.89] | 1.52 [1.32, 1.73] | .66 | 142.58 [104.11, 187.77] | 138.73 [102.07, 165.64] | .39 |
| SCGB3A2 | 0.80 [0.66, 0.92] | 0.74 [0.68, 0.86] | .53 | 3.05 [2.01, 5.45] | 2.52 [1.93, 3.58] | .34 |
| EGFR | 0.78 [0.72, 0.83] | 0.76 [0.71, 0.86] | 1.0 | 4.38 [3.71, 5.03] | 4.23 [3.40, 4.92] | .57 |
| IGFBP7 | 2.49 [2.19, 3.24] | 2.53 [2.25, 3.51] | .39 | 132.87 [106.54, 165.98] | 115.58 [88.68, 194.35] | .53 |
| CD93 | 34.01 [27.25, 45.50] | 32.71 [30.17, 47.99] | .52 | 1524.89 [1147.99, 1866.83] | 1275.94 [972.37, 2030.87] | .65 |
| IL18BP | 1.92 [1.67, 2.26] | 1.95 [1.53, 2.34] | .81 | 51.01 [42.57, 63.22] | 48.74 [37.15, 63.24] | .87 |
| COL1A1 | 0.75 [0.72, 0.78] | 0.75 [0.73, 0.78] | .68 | 4.38 [3.78, 5.59] | 4.23 [3.56, 6.08] | .97 |
| PON3 | 1.53 [1.33, 1.89] | 1.67 [1.33, 1.85] | .79 | 28.78 [21.58, 38.58] | 29.03 [19.79, 41.74] | .97 |
| CTSZ | 1.13 [1.04, 1.27] | 1.09 [1.01, 1.38] | .94 | 29.92 [23.31, 36.19] | 25.63 [22.00, 37.81] | .43 |
| MMP3 | 1.87 [1.28, 2.47] | 2.03 [1.86, 2.16] | .48 | 119.31 [86.99, 202.71] | 127.78 [91.02, 150.15] | .73 |
| RARRES2 | 7.81 [6.30, 9.70] | 8.43 [6.91, 10.33] | .19 | 1858.91 [1564.04, 2216.59] | 1836.87 [1460.44, 2108.66] | .80 |
| ICAM2 | 2.30 [1.90, 2.67] | 2.35 [2.09, 2.66] | .65 | 23.83 [19.51, 31.87] | 24.41 [19.35, 32.65] | .89 |
| KLK6 | 0.23 [0.19, 0.31] | 0.22 [0.19, 0.25] | .47 | 3.63 [2.96, 4.24] | 3.27 [2.53, 3.58] | .038 |
| PDGFsubunitA | 1.02 [0.97, 1.11] | 1.06 [1.01, 1.12] | .13 | 3.14 [2.48, 3.81] | 2.43 [2.16, 3.11] | .035 |
| TNFR1 | 3.31 [2.90, 3.85] | 3.48 [2.83, 4.92] | .43 | 67.62 [52.24, 83.26] | 70.14 [49.68, 110.51] | .61 |
| IGFBP2 | 3.45 [2.68, 4.63] | 4.64 [2.57, 5.69] | .21 | 207.49 [141.64, 291.93] | 184.08 [148.90, 265.82] | .62 |
| vWF | 2.96 [2.48, 4.02] | 3.38 [2.45, 4.41] | .67 | 135.04 [102.86, 244.11] | 175.68 [125.45, 217.37] | .81 |
| PECAM1 | 0.72 [0.62, 0.82] | 0.75 [0.66, 0.80] | .58 | 13.88 [12.00, 17.59] | 13.21 [10.76, 18.52] | .54 |
| MEPE | 1.03 [0.81, 1.20] | 1.25 [1.00, 1.42] | .004 | 21.60 [18.01, 27.63] | 19.99 [15.79, 27.33] | .53 |
| CCL16 | 1.37 [1.15, 1.75] | 1.31 [1.07, 1.60] | .57 | 66.24 [53.72, 78.74] | 55.34 [42.99, 73.38] | .06 |

|  | *Plaque* | | |
| --- | --- | --- | --- |
|  | *No Stroke* | *Stroke* | *P* |
|  | N = 67 | N =21 |  |
| TNFRSF14 | 3.41 [2.99, 4.28] | 3.82 [3.16, 4.43] | .51 |
| LDLreceptor | 0.54 [0.49, 0.59] | 0.57 [0.50, 0.62] | .27 |
| ITGB2 | 3.32 [1.99, 5.29] | 5.49 [3.90, 9.87] | .002 |
| IL17RA | 1.31 [1.11, 1.59] | 1.47 [1.20, 1.64] | .40 |
| TNFR2 | 3.28 [2.63, 4.22] | 3.98 [3.15, 4.21] | .12 |
| MMP9 | 1.29 [1.03, 1.96] | 3.39 [1.92, 5.04] | <.001 |
| EPHB4 | 1.75 [1.48, 2.14] | 1.86 [1.67, 2.02] | .81 |
| IL2RA | 1.05 [0.94, 1.21] | 1.07 [0.95, 1.23] | .98 |
| OPG | 15.99 [9.35, 28.71] | 20.38 [10.95, 25.44] | .40 |
| ALCAM | 4.07 [2.83, 6.25] | 4.04 [3.44, 7.84] | .58 |
| TFF3 | 1.19 [1.03, 1.43] | 1.16 [1.05, 1.23] | .60 |
| SELP | 7.73 [5.91, 11.52] | 7.16 [6.43, 8.50] | .51 |
| CSTB | 110.50 [56.35, 167.74] | 181.78 [86.77, 271.58] | .070 |
| MCP1 | 4.90 [2.71, 8.67] | 7.31 [5.40, 12.76] | .032 |
| CD163 | 8.32 [4.76, 15.73] | 15.73 [8.67, 24.22] | .015 |
| Gal3 | 1.12 [0.58, 1.89] | 1.42 [0.94, 1.90] | .39 |
| GRN | 1.96 [1.36, 2.58] | 2.76 [1.95, 3.66] | .010 |
| NTproBNP | 2.80 [2.58, 3.15] | 2.68 [2.46, 2.81] | .062 |
| BLMhydrolase | 1.21 [0.77, 1.99] | 1.50 [1.07, 1.70] | .34 |
| PLC | 11.88 [5.47, 23.67] | 15.82 [7.12, 19.06] | .71 |
| LTBR | 1.16 [0.91, 1.36] | 1.06 [0.85, 1.33] | .89 |
| Notch3 | 1.42 [1.18, 1.64] | 1.28 [1.24, 1.68] | .93 |
| TIMP4 | 0.70 [0.63, 0.81] | 0.70 [0.62, 0.76] | .66 |
| CNTN1 | 0.58 [0.53, 0.64] | 0.56 [0.53, 0.59] | .30 |
| CDH5 | 1.98 [1.72, 2.34] | 1.89 [1.66, 2.14] | .67 |
| TLT2 | 2.63 [2.19, 3.32] | 2.53 [2.03, 2.90] | .12 |
| FABP4 | 11.33 [4.55, 20.74] | 24.32 [6.15, 50.33] | .10 |
| TFPI | 4.23 [3.31, 6.14] | 4.71 [4.26, 5.25] | .49 |
| PAI | 2.85 [1.97, 5.07] | 3.91 [2.95, 5.08] | .16 |
| CCL24 | 0.75 [0.59, 0.95] | 0.80 [0.69, 0.92] | .61 |
| TR | 0.87 [0.75, 1.12] | 1.05 [0.86, 1.34] | .018 |
| TNFRSF10C | 2.89 [2.35, 3.56] | 2.90 [2.32, 4.64] | .75 |
| GDF15 | 2.92 [2.16, 4.37] | 3.63 [3.25, 9.53] | .003 |
| SELE | 12.70 [8.95, 18.22] | 14.02 [10.35, 18.24] | .46 |
| AZU1 | 2.18 [1.30, 4.13] | 4.26 [2.11, 6.62] | .024 |
| DLK1 | 1.49 [1.35, 1.82] | 1.43 [1.25, 1.64] | .18 |
| SPON1 | 2.20 [1.98, 2.62] | 2.15 [1.96, 2.49] | .33 |
| MPO | 1.44 [1.07, 3.10] | 3.71 [1.70, 6.56] | .012 |
| CXCL16 | 2.09 [1.64, 2.95] | 2.53 [2.19, 3.30] | .011 |
| IL6RA | 13.51 [9.80, 17.29] | 15.67 [11.97, 21.21] | .29 |
| RETN | 2.98 [2.16, 5.50] | 4.60 [2.91, 7.50] | .099 |
| IGFBP1 | 1.90 [1.67, 2.32] | 1.97 [1.76, 2.19] | .84 |
| CHIT1 | 1.62 [1.25, 2.68] | 1.97 [1.45, 3.06] | .30 |
| TRAP | 1.05 [0.51, 1.49] | 1.69 [0.97, 2.41] | .022 |
| GP6 | 0.73 [0.68, 0.83] | 0.71 [0.63, 0.86] | .48 |
| PSPD | 0.79 [0.69, 1.04] | 0.75 [0.64, 1.08] | .71 |
| PI3 | 0.26 [0.22, 0.32] | 0.24 [0.22, 0.29] | .52 |
| EpCAM | 1.17 [0.90, 1.53] | 1.22 [0.91, 1.51] | .85 |
| APN | 1.00 [0.89, 1.21] | 1.14 [0.97, 1.30] | .065 |
| AXL | 5.44 [4.32, 6.68] | 5.65 [4.14, 6.38] | .83 |
| IL1RT1 | 1.77 [1.48, 2.20] | 1.83 [1.49, 2.06] | 1.0 |
| MMP2 | 0.79 [0.74, 0.84] | 0.79 [0.75, 0.82] | .77 |
| FAS | 1.46 [1.02, 2.23] | 1.29 [1.14, 1.71] | .42 |
| MB | 9.70 [4.13, 19.73] | 3.91 [2.62, 16.39] | .12 |
| TNFSF13B | 3.10 [2.34, 4.15] | 3.62 [2.73, 4.40] | .22 |
| PRTN3 | 1.17 [0.86, 1.56] | 1.47 [1.18, 3.00] | .024 |
| PCSK9 | 1.12 [0.97, 1.28] | 1.10 [0.97, 1.17] | .41 |
| UPAR | 10.64 [5.23, 23.46] | 25.08 [13.03, 48.99] | .001 |
| OPN | 20.78 [9.19, 54.89] | 52.36 [15.42, 116.12] | .090 |
| CTSD | 5.45 [1.37, 10.69] | 8.09 [1.97, 15.60] | .30 |
| PGLYRP1 | 2.49 [1.87, 4.07] | 3.63 [2.33, 5.44] | .085 |
| CPA1 | 0.97 [0.81, 1.16] | 0.88 [0.78, 1.03] | .32 |
| JAMA | 0.65 [0.54, 0.97] | 0.83 [0.63, 0.98] | .29 |
| Gal4 | 1.05 [0.91, 1.27] | 1.04 [0.92, 1.15] | .59 |
| IL1RT2 | 0.99 [0.85, 1.20] | 1.04 [0.91, 1.10] | .74 |
| SHPS1 | 1.68 [1.42, 1.94] | 1.65 [1.43, 2.09] | .77 |
| CCL15 | 1.61 [1.42, 1.88] | 1.38 [1.26, 1.77] | .17 |
| CASP3 | 27.80 [9.73, 44.27] | 41.84 [24.93, 59.60] | .016 |
| uPA | 1.72 [1.12, 2.80] | 3.10 [2.16, 4.19] | .003 |
| CPB1 | 0.87 [0.79, 1.00] | 0.90 [0.84, 0.99] | .65 |
| CHI3L1 | 0.60 [0.36, 1.08] | 1.31 [0.68, 1.98] | .014 |
| ST2 | 2.29 [1.87, 2.62] | 2.12 [1.83, 2.40] | .40 |
| tPA | 2.41 [1.94, 3.32] | 2.48 [2.01, 3.06] | .83 |
| SCGB3A2 | 0.70 [0.62, 0.77] | 0.68 [0.64, 0.74] | .97 |
| EGFR | 0.96 [0.90, 1.00] | 0.94 [0.92, 0.97] | .69 |
| IGFBP7 | 50.73 [19.42, 105.78] | 44.35 [30.02, 64.23] | .75 |
| CD93 | 16.49 [10.36, 23.76] | 19.32 [14.47, 25.45] | .41 |
| IL18BP | 1.63 [1.37, 2.22] | 2.25 [1.82, 2.62] | .002 |
| COL1A1 | 1.05 [0.94, 1.26] | 1.10 [1.03, 1.60] | .25 |
| PON3 | 1.25 [1.12, 1.40] | 1.19 [1.07, 1.32] | .18 |
| CTSZ | 8.13 [5.15, 13.41] | 13.72 [6.80, 17.96] | .049 |
| MMP3 | 2.13 [1.82, 2.45] | 2.02 [1.40, 3.02] | .64 |
| RARRES2 | 5.20 [2.71, 10.24] | 3.88 [2.41, 4.44] | .067 |
| ICAM2 | 2.44 [2.18, 2.80] | 2.31 [2.13, 2.54] | .22 |
| KLK6 | 0.29 [0.22, 0.37] | 0.29 [0.23, 0.37] | .97 |
| PDGFsubunitA | 1.48 [1.36, 1.58] | 1.44 [1.33, 1.51] | .21 |
| TNFR1 | 6.98 [5.78, 8.51] | 7.76 [6.84, 10.67] | .12 |
| IGFBP2 | 9.40 [5.73, 15.76] | 8.71 [6.90, 12.40] | .85 |
| vWF | 2.00 [1.57, 2.46] | 2.17 [1.85, 2.64] | .15 |
| PECAM1 | 1.09 [0.89, 1.27] | 1.07 [0.95, 1.20] | .98 |
| MEPE | 1.37 [1.22, 1.64] | 1.36 [1.23, 1.54] | .77 |
| CCL16 | 0.83 [0.72, 1.07] | 0.77 [0.71, 0.92] | .49 |

### Supplementary Tables 2A-B:

For the following 2 tables, EV, plasma and plaque proteins are stratified for three-year major adverse cardiovascular event. These proteins are measured with the Olink Cardiovascular III panel. Non-parametric Mann-Whitney U test is performed with protein expression given as median [interquartile range].

For the abbreviations of proteins, we refer to Olink’s website: <https://www.olink.com/content/uploads/2021/09/1023-v1.3-cvd-iii-panel-content-final.pdf>

|  | *EV* | | | *Plasma* | | |
| --- | --- | --- | --- | --- | --- | --- |
|  | *No MACE* | *MACE* | *P* | *No MACE* | *MACE* | *P* |
|  | N = 66 | N = 22 |  | N = 66 | N = 22 |  |
| TNFRSF14 | 1.56 [1.33, 1.85] | 1.81 [1.64, 2.10] | .013 | 17.65 [14.47, 21.82] | 22.49 [16.42, 25.65] | .031 |
| LDLreceptor | 0.42 [0.34, 0.50] | 0.41 [0.38, 0.52] | .77 | 7.53 [5.76, 10.65] | 8.34 [6.73, 11.26] | .68 |
| ITGB2 | 0.94 [0.77, 1.14] | 1.03 [0.93, 1.10] | .410 | 15.05 [12.29, 19.61] | 15.12 [13.36, 19.12] | .58 |
| IL17RA | 0.97 [0.76, 1.08] | 1.02 [0.92, 1.18] | .086 | 7.81 [6.30, 10.64] | 9.84 [8.24, 11.99] | .072 |
| TNFR2 | 2.31 [1.85, 2.75] | 2.57 [2.16, 3.14] | .066 | 25.70 [21.97, 33.80] | 32.76 [22.12, 47.98] | .080 |
| MMP9 | 0.70 [0.62, 0.84] | 0.78 [0.69, 0.96] | .036 | 10.17 [6.45, 14.30] | 11.17 [6.64, 17.25] | .546 |
| EPHB4 | 1.82 [1.53, 2.03] | 1.94 [1.66, 2.27] | .244 | 25.95 [22.05, 30.87] | 29.09 [24.61, 40.79] | .052 |
| IL2RA | 0.70 [0.63, 0.78] | 0.70 [0.66, 0.86] | .254 | 10.09 [7.49, 12.18] | 10.37 [8.38, 14.58] | .47 |
| OPG | 0.80 [0.71, 0.92] | 0.91 [0.73, 1.00] | .103 | 11.98 [9.49, 14.32] | 13.20 [9.90, 15.75] | .17 |
| ALCAM | 3.15 [2.62, 3.63] | 3.37 [2.97, 3.89] | .108 | 108.37 [85.79, 129.28] | 110.68 [99.62, 127.13] | .57 |
| TFF3 | 1.40 [1.14, 1.60] | 1.60 [1.41, 1.93] | .029 | 22.88 [17.04, 27.36] | 26.48 [23.66, 35.32] | .021 |
| SELP | 12.65 [9.89, 17.78] | 15.69 [11.79, 20.02] | .032 | 513.28 [410.79, 729.79] | 619.12 [533.12, 736.13] | .090 |
| CSTB | 0.53 [0.46, 0.61] | 0.57 [0.49, 0.64] | .230 | 9.54 [7.56, 12.45] | 11.72 [6.95, 15.39] | .15 |
| MCP1 | 0.98 [0.76, 1.07] | 1.01 [0.83, 1.22] | .234 | 14.06 [10.33, 17.27] | 14.31 [10.73, 19.32] | .37 |
| CD163 | 3.72 [2.94, 4.71] | 3.76 [3.28, 4.36] | .996 | 114.25 [97.65, 157.43] | 107.24 [101.02, 130.00] | .62 |
| Gal3 | 0.23 [0.20, 0.26] | 0.22 [0.20, 0.24] | .670 | 6.62 [5.43, 8.51] | 7.23 [6.34, 8.70] | .31 |
| GRN | 1.27 [1.15, 1.36] | 1.38 [1.25, 1.48] | .049 | 29.37 [23.82, 32.80] | 30.84 [25.27, 34.46] | .35 |
| NTproBNP | 2.16 [1.77, 2.47] | 2.37 [2.11, 2.71] | .052 | 12.46 [5.56, 19.56] | 20.84 [12.50, 29.66] | .004 |
| BLMhydrolase | 0.20 [0.18, 0.22] | 0.20 [0.17, 0.22] | .417 | 2.87 [2.58, 3.38] | 2.52 [2.21, 3.40] | .25 |
| PLC | 4.26 [3.53, 5.53] | 5.02 [3.97, 7.01] | .077 | 203.15 [160.22, 270.72] | 221.68 [199.28, 299.30] | .070 |
| LTBR | 0.55 [0.45, 0.62] | 0.65 [0.57, 0.69] | .005 | 7.54 [6.07, 9.32] | 8.65 [6.98, 12.06] | .070 |
| Notch3 | 1.04 [0.86, 1.16] | 1.10 [0.99, 1.36] | .091 | 21.34 [16.28, 29.42] | 22.33 [17.74, 27.60] | .71 |
| TIMP4 | 0.53 [0.47, 0.64] | 0.59 [0.52, 0.67] | .130 | 9.40 [7.60, 11.31] | 10.12 [7.49, 15.12] | .22 |
| CNTN1 | 0.40 [0.36, 0.46] | 0.40 [0.35, 0.47] | .747 | 9.27 [7.55, 11.39] | 10.45 [6.87, 11.22] | .98 |
| CDH5 | 1.46 [1.24, 1.68] | 1.51 [1.37, 1.73] | .379 | 11.77 [9.34, 14.37] | 12.51 [9.65, 15.33] | .34 |
| TLT2 | 2.07 [1.65, 2.71] | 2.18 [1.83, 2.50] | .992 | 17.76 [13.88, 21.59] | 20.97 [16.23, 22.60] | .23 |
| FABP4 | 1.11 [0.97, 1.47] | 1.48 [1.04, 1.87] | .099 | 36.24 [26.98, 53.19] | 32.03 [26.69, 69.36] | .75 |
| TFPI | 9.89 [7.48, 11.33] | 9.58 [7.97, 11.87] | .699 | 385.09 [276.67, 446.25] | 371.54 [294.84, 443.15] | .77 |
| PAI | 0.58 [0.51, 0.73] | 0.62 [0.57, 0.75] | .383 | 14.45 [10.55, 24.17] | 16.48 [10.92, 19.90] | .78 |
| CCL24 | 0.68 [0.52, 0.81] | 0.75 [0.56, 0.93] | .180 | 19.81 [13.86, 32.18] | 20.98 [15.19, 34.44] | .50 |
| TR | 1.45 [1.21, 1.69] | 1.43 [1.30, 1.87] | .605 | 13.65 [10.79, 19.39] | 16.83 [11.78, 22.94] | .22 |
| TNFRSF10C | 2.96 [2.59, 4.04] | 3.43 [2.65, 4.24] | .261 | 55.75 [41.30, 70.48] | 60.03 [50.97, 81.79] | .27 |
| GDF15 | 1.16 [0.98, 1.50] | 1.49 [1.18, 2.15] | .011 | 46.43 [33.84, 61.59] | 68.99 [47.95, 100.28] | .005 |
| SELE | 73.66 [52.00, 91.60] | 91.86 [59.87, 119.25] | .204 | 2358.77 [1724.27, 3077.42] | 2496.39 [2143.09, 3517.78] | .23 |
| AZU1 | 0.28 [0.24, 0.33] | 0.26 [0.24, 0.30] | .570 | 4.65 [3.07, 7.73] | 4.56 [3.26, 7.94] | .95 |
| DLK1 | 1.89 [1.56, 2.43] | 2.35 [1.84, 2.54] | .044 | 39.38 [27.82, 59.59] | 52.42 [34.42, 63.95] | .18 |
| SPON1 | 1.50 [1.30, 1.76] | 1.64 [1.47, 1.72] | .123 | 2.29 [1.97, 2.66] | 2.48 [2.27, 2.92] | .075 |
| MPO | 0.60 [0.52, 0.65] | 0.62 [0.56, 0.69] | .184 | 7.24 [5.28, 9.70] | 7.76 [6.31, 9.32] | .42 |
| CXCL16 | 1.51 [1.25, 1.78] | 1.84 [1.31, 2.08] | .066 | 27.20 [20.76, 35.14] | 31.17 [25.03, 35.96] | .21 |
| IL6RA | 48.14 [36.58, 63.37] | 50.62 [43.49, 52.70] | .945 | 2984.55 [2265.78, 3573.98] | 3267.84 [2426.32, 4320.49] | .19 |
| RETN | 0.97 [0.80, 1.26] | 1.21 [0.92, 1.47] | .058 | 61.15 [43.97, 74.97] | 69.50 [56.77, 84.00] | .072 |
| IGFBP1 | 1.11 [0.90, 1.28] | 1.38 [0.97, 1.44] | .061 | 18.51 [11.68, 35.26] | 14.48 [8.55, 32.20] | .49 |
| CHIT1 | 1.39 [1.13, 1.68] | 1.66 [1.29, 1.98] | .083 | 27.80 [18.47, 38.98] | 33.85 [24.25, 54.16] | .076 |
| TRAP | 0.33 [0.28, 0.37] | 0.31 [0.28, 0.35] | .847 | 7.52 [5.81, 9.06] | 7.19 [5.59, 8.91] | .81 |
| GP6 | 0.44 [0.40, 0.52] | 0.49 [0.44, 0.57] | .056 | 1.60 [1.38, 2.04] | 1.91 [1.60, 2.52] | .091 |
| PSPD | 0.58 [0.48, 0.66] | 0.67 [0.54, 0.81] | .069 | 3.77 [2.32, 6.27] | 5.00 [2.79, 6.26] | .37 |
| PI3 | 0.33 [0.30, 0.39] | 0.34 [0.30, 0.38] | .682 | 2.38 [1.93, 3.50] | 3.34 [2.62, 4.53] | .014 |
| EpCAM | 1.21 [0.99, 1.68] | 1.22 [0.91, 2.05] | .751 | 20.84 [13.80, 42.99] | 26.62 [12.44, 45.22] | .65 |
| APN | 1.01 [0.89, 1.18] | 1.13 [0.95, 1.18] | .283 | 20.81 [15.73, 25.77] | 21.81 [17.96, 24.23] | .52 |
| AXL | 7.65 [6.25, 8.89] | 7.63 [6.61, 8.02] | .743 | 214.74 [182.35, 266.95] | 206.28 [183.47, 254.94] | .59 |
| IL1RT1 | 1.81 [1.47, 2.06] | 1.59 [1.51, 1.96] | .531 | 46.27 [38.51, 52.80] | 46.44 [37.40, 55.22] | .82 |
| MMP2 | 0.60 [0.56, 0.65] | 0.63 [0.58, 0.71] | .072 | 6.87 [5.21, 8.36] | 7.32 [5.77, 8.34] | .64 |
| FAS | 0.84 [0.72, 0.95] | 0.94 [0.83, 1.08] | .057 | 28.60 [21.47, 35.32] | 32.02 [26.92, 36.56] | .13 |
| MB | 3.31 [2.58, 4.53] | 3.41 [2.86, 5.26] | .419 | 112.28 [80.04, 163.66] | 99.27 [79.32, 167.30] | .96 |
| TNFSF13B | 2.97 [2.44, 3.79] | 3.11 [2.62, 3.58] | .782 | 86.85 [64.05, 127.22] | 88.53 [74.52, 100.28] | .48 |
| PRTN3 | 0.43 [0.36, 0.53] | 0.43 [0.37, 0.53] | .767 | 7.98 [5.62, 11.32] | 8.41 [6.35, 11.62] | .58 |
| PCSK9 | 0.79 [0.70, 0.97] | 0.94 [0.81, 1.06] | .022 | 4.58 [3.58, 5.84] | 4.72 [4.19, 6.00] | .34 |
| UPAR | 1.68 [1.35, 1.94] | 1.90 [1.67, 2.13] | .010 | 28.25 [20.96, 34.33] | 32.03 [28.39, 38.78] | .045 |
| OPN | 0.69 [0.60, 0.82] | 0.83 [0.73, 0.98] | .004 | 92.72 [76.81, 130.45] | 127.78 [82.73, 210.96] | .054 |
| CTSD | 0.25 [0.22, 0.28] | 0.25 [0.22, 0.32] | .566 | 5.59 [3.99, 6.41] | 5.92 [4.20, 7.85] | .30 |
| PGLYRP1 | 2.18 [1.62, 2.49] | 2.28 [1.76, 3.02] | .172 | 106.78 [85.89, 148.83] | 127.88 [97.21, 171.84] | .16 |
| CPA1 | 0.98 [0.77, 1.23] | 1.18 [0.94, 1.53] | .053 | 33.30 [21.81, 47.96] | 36.33 [28.01, 53.27] | .30 |
| JAMA | 0.44 [0.36, 0.55] | 0.53 [0.43, 0.62] | .024 | 10.33 [7.96, 13.05] | 13.50 [10.61, 14.81] | .010 |
| Gal4 | 0.81 [0.69, 0.90] | 0.93 [0.82, 1.16] | .006 | 9.13 [7.23, 12.76] | 10.64 [8.56, 15.87] | .12 |
| IL1RT2 | 0.99 [0.80, 1.20] | 1.08 [0.92, 1.28] | .114 | 21.75 [18.54, 27.04] | 23.37 [20.95, 26.58] | .36 |
| SHPS1 | 0.86 [0.77, 0.93] | 0.96 [0.86, 1.03] | .011 | 7.13 [5.79, 8.65] | 7.12 [4.98, 10.96] | .77 |
| CCL15 | 2.00 [1.56, 2.19] | 2.12 [1.73, 3.03] | .036 | 108.60 [81.89, 128.65] | 126.34 [103.50, 179.44] | .013 |
| CASP3 | 0.89 [0.68, 1.31] | 1.13 [0.81, 1.36] | .210 | 30.05 [20.22, 40.04] | 29.34 [18.82, 71.72] | .52 |
| uPA | 0.59 [0.51, 0.69] | 0.66 [0.58, 0.75] | .061 | 16.34 [12.39, 20.77] | 16.82 [14.82, 22.03] | .23 |
| CPB1 | 0.83 [0.73, 0.96] | 0.97 [0.76, 1.09] | .069 | 28.78 [18.60, 44.49] | 32.25 [23.83, 58.77] | .20 |
| CHI3L1 | 0.32 [0.22, 0.56] | 0.35 [0.28, 0.54] | .487 | 10.92 [6.86, 19.91] | 14.03 [10.42, 20.05] | .16 |
| ST2 | 1.60 [1.21, 1.96] | 1.71 [1.36, 2.16] | .166 | 12.85 [10.57, 16.62] | 16.62 [13.65, 22.49] | .018 |
| tPA | 1.51 [1.24, 1.90] | 1.55 [1.44, 1.80] | .292 | 132.62 [100.50, 178.26] | 157.83 [121.82, 198.72] | .14 |
| SCGB3A2 | 0.77 [0.65, 0.92] | 0.74 [0.70, 0.86] | .744 | 3.05 [1.59, 5.01] | 2.64 [2.14, 5.32] | .82 |
| EGFR | 0.78 [0.71, 0.84] | 0.79 [0.74, 0.87] | .187 | 4.41 [3.60, 5.00] | 4.17 [3.79, 5.16] | .89 |
| IGFBP7 | 2.48 [2.19, 3.06] | 2.82 [2.37, 3.60] | .042 | 128.19 [94.28, 169.28] | 138.67 [107.96, 170.89] | .28 |
| CD93 | 32.51 [27.37, 45.42] | 40.12 [33.82, 50.94] | .055 | 1455.41 [1054.18, 1841.00] | 1645.75 [1289.52, 2170.66] | .11 |
| IL18BP | 1.83 [1.60, 2.21] | 2.23 [1.87, 2.44] | .033 | 48.04 [38.36, 61.66] | 52.35 [48.23, 72.92] | .17 |
| COL1A1 | 0.75 [0.72, 0.78] | 0.75 [0.72, 0.81] | .374 | 4.23 [3.64, 5.59] | 4.67 [3.73, 5.90] | .53 |
| PON3 | 1.53 [1.33, 1.84] | 1.63 [1.41, 1.97] | .571 | 27.98 [21.03, 38.80] | 30.14 [24.22, 41.71] | .50 |
| CTSZ | 1.11 [1.01, 1.27] | 1.17 [1.07, 1.39] | .214 | 29.61 [22.88, 36.66] | 27.43 [23.66, 41.08] | .70 |
| MMP3 | 1.87 [1.27, 2.40] | 2.18 [1.62, 2.45] | .257 | 110.24 [89.90, 187.68] | 132.02 [88.45, 195.15] | .48 |
| RARRES2 | 7.76 [6.31, 9.33] | 8.81 [7.63, 13.16] | .036 | 1832.44 [1456.76, 2132.89] | 1964.53 [1727.83, 2359.37] | .092 |
| ICAM2 | 2.30 [1.88, 2.61] | 2.33 [2.08, 2.92] | .177 | 23.67 [19.36, 31.05] | 26.81 [19.84, 33.45] | .41 |
| KLK6 | 0.23 [0.19, 0.31] | 0.22 [0.19, 0.26] | .236 | 3.47 [2.75, 4.09] | 3.56 [3.01, 4.20] | .50 |
| PDGFsubunitA | 1.02 [0.97, 1.11] | 1.05 [0.99, 1.17] | .340 | 2.84 [2.37, 3.80] | 3.12 [2.44, 3.72] | .56 |
| TNFR1 | 3.16 [2.78, 3.81] | 3.94 [3.07, 4.99] | .011 | 67.44 [50.88, 81.78] | 78.76 [58.50, 111.35] | .077 |
| IGFBP2 | 3.45 [2.59, 4.54] | 4.85 [3.03, 5.85] | .049 | 193.02 [134.34, 268.79] | 228.95 [159.01, 296.82] | .30 |
| vWF | 2.83 [2.43, 4.09] | 3.31 [2.76, 4.39] | .157 | 135.49 [106.28, 213.69] | 198.74 [98.11, 250.20] | .34 |
| PECAM1 | 0.71 [0.63, 0.79] | 0.75 [0.67, 0.84] | .254 | 13.44 [11.32, 17.27] | 15.19 [13.70, 20.13] | .058 |
| MEPE | 1.03 [0.83, 1.20] | 1.19 [0.96, 1.38] | .130 | 20.37 [16.51, 27.70] | 23.48 [19.53, 26.93] | .25 |
| CCL16 | 1.35 [1.10, 1.66] | 1.48 [1.27, 1.97] | .047 | 62.51 [47.10, 75.85] | 62.90 [54.95, 92.32] | .26 |

|  | *Plaque* | | |
| --- | --- | --- | --- |
|  | *No MACE* | *MACE* | *P* |
|  | 66 | 22 |  |
| TNFRSF14 | 3.45 [2.99, 4.29] | 3.48 [3.03, 4.15] | .84 |
| LDLreceptor | 0.55 [0.49, 0.60] | 0.53 [0.49, 0.59] | .54 |
| ITGB2 | 3.84 [2.26, 6.84] | 3.38 [1.74, 5.75] | .27 |
| IL17RA | 1.34 [1.13, 1.59] | 1.33 [1.17, 1.57] | .77 |
| TNFR2 | 3.56 [2.62, 4.27] | 3.36 [3.04, 4.14] | .93 |
| MMP9 | 1.46 [1.09, 2.78] | 1.62 [1.03, 2.51] | .99 |
| EPHB4 | 1.79 [1.49, 2.08] | 1.92 [1.66, 2.18] | .26 |
| IL2RA | 1.05 [0.94, 1.22] | 1.07 [0.97, 1.19] | .79 |
| OPG | 19.43 [9.57, 27.15] | 13.21 [10.70, 29.40] | .42 |
| ALCAM | 4.07 [2.95, 6.93] | 3.35 [2.65, 5.67] | .27 |
| TFF3 | 1.16 [1.03, 1.30] | 1.21 [1.04, 1.50] | .37 |
| SELP | 7.65 [6.08, 10.29] | 7.23 [5.65, 11.73] | .89 |
| CSTB | 114.71 [67.94, 204.86] | 103.73 [48.86, 181.30] | .32 |
| MCP1 | 5.55 [2.87, 9.67] | 4.88 [2.88, 10.05] | .71 |
| CD163 | 10.23 [6.26, 18.84] | 7.47 [4.86, 15.53] | .23 |
| Gal3 | 1.23 [0.63, 1.90] | 1.18 [0.58, 1.89] | .60 |
| GRN | 2.11 [1.63, 2.90] | 1.69 [1.28, 2.78] | .14 |
| NTproBNP | 2.78 [2.58, 3.12] | 2.78 [2.57, 2.95] | .66 |
| BLMhydrolase | 1.37 [0.91, 1.93] | 0.99 [0.78, 1.71] | .30 |
| PLC | 13.39 [7.44, 22.72] | 12.14 [5.16, 21.38] | .44 |
| LTBR | 1.15 [0.93, 1.34] | 0.98 [0.84, 1.42] | .58 |
| Notch3 | 1.34 [1.18, 1.54] | 1.49 [1.27, 1.73] | .11 |
| TIMP4 | 0.71 [0.63, 0.81] | 0.70 [0.65, 0.74] | .63 |
| CNTN1 | 0.57 [0.53, 0.62] | 0.56 [0.51, 0.66] | 1.0 |
| CDH5 | 1.97 [1.72, 2.26] | 1.98 [1.68, 2.21] | .82 |
| TLT2 | 2.59 [2.15, 3.30] | 2.80 [2.17, 3.00] | .94 |
| FABP4 | 10.85 [4.89, 27.58] | 12.85 [5.72, 25.32] | .48 |
| TFPI | 4.44 [3.52, 6.15] | 4.17 [3.22, 5.40] | .32 |
| PAI | 3.42 [2.13, 5.44] | 2.97 [2.02, 4.11] | .34 |
| CCL24 | 0.72 [0.59, 0.94] | 0.80 [0.70, 0.96] | .19 |
| TR | 0.92 [0.78, 1.22] | 0.88 [0.82, 1.14] | .87 |
| TNFRSF10C | 2.89 [2.34, 3.83] | 2.94 [2.07, 3.68] | .82 |
| GDF15 | 3.10 [2.22, 5.09] | 3.20 [2.43, 4.57] | .93 |
| SELE | 12.70 [9.82, 17.95] | 14.16 [9.38, 18.02] | .95 |
| AZU1 | 2.67 [1.20, 5.00] | 2.20 [1.75, 3.61] | .92 |
| DLK1 | 1.45 [1.32, 1.79] | 1.45 [1.40, 1.64] | .60 |
| SPON1 | 2.19 [1.96, 2.55] | 2.33 [2.07, 2.54] | .51 |
| MPO | 1.66 [1.07, 4.09] | 1.87 [1.20, 3.46] | .91 |
| CXCL16 | 2.27 [1.72, 3.17] | 2.22 [2.03, 2.69] | .98 |
| IL6RA | 13.76 [9.00, 18.18] | 14.04 [11.27, 19.47] | .61 |
| RETN | 3.44 [2.38, 5.86] | 2.97 [2.20, 5.39] | .70 |
| IGFBP1 | 1.90 [1.56, 2.30] | 1.97 [1.76, 2.26] | .39 |
| CHIT1 | 1.89 [1.28, 3.02] | 1.69 [1.20, 2.61] | .48 |
| TRAP | 1.17 [0.56, 1.85] | 1.12 [0.42, 1.78] | .59 |
| GP6 | 0.72 [0.63, 0.83] | 0.75 [0.70, 0.91] | .079 |
| PSPD | 0.75 [0.62, 1.04] | 0.84 [0.72, 1.20] | .15 |
| PI3 | 0.26 [0.22, 0.30] | 0.26 [0.22, 0.33] | .79 |
| EpCAM | 1.15 [0.90, 1.51] | 1.27 [1.03, 1.58] | .23 |
| APN | 1.01 [0.89, 1.28] | 1.02 [0.91, 1.13] | .85 |
| AXL | 5.28 [4.24, 6.60] | 6.08 [4.73, 6.99] | .36 |
| IL1RT1 | 1.82 [1.43, 2.21] | 1.67 [1.52, 2.15] | .70 |
| MMP2 | 0.78 [0.73, 0.82] | 0.81 [0.76, 0.85] | .097 |
| FAS | 1.50 [1.14, 2.06] | 1.24 [1.02, 1.94] | .25 |
| MB | 8.21 [3.60, 20.01] | 6.48 [2.90, 15.38] | .36 |
| TNFSF13B | 3.37 [2.48, 4.32] | 3.09 [2.04, 4.07] | .21 |
| PRTN3 | 1.19 [0.92, 1.81] | 1.33 [0.96, 1.76] | .73 |
| PCSK9 | 1.09 [0.97, 1.20] | 1.19 [0.96, 1.35] | .14 |
| UPAR | 15.26 [6.40, 28.52] | 8.72 [6.10, 20.86] | .32 |
| OPN | 23.25 [10.05, 80.42] | 23.14 [11.47, 71.69] | .97 |
| CTSD | 6.05 [1.63, 12.50] | 6.01 [1.44, 9.24] | .46 |
| PGLYRP1 | 2.62 [1.94, 4.55] | 2.60 [2.00, 3.62] | .88 |
| CPA1 | 0.91 [0.78, 1.07] | 1.00 [0.89, 1.21] | .054 |
| JAMA | 0.71 [0.55, 1.01] | 0.62 [0.51, 0.81] | .22 |
| Gal4 | 1.05 [0.92, 1.22] | 1.10 [0.93, 1.35] | .46 |
| IL1RT2 | 1.00 [0.85, 1.12] | 1.00 [0.90, 1.29] | .74 |
| SHPS1 | 1.75 [1.45, 2.01] | 1.55 [1.41, 1.83] | .27 |
| CCL15 | 1.59 [1.34, 1.82] | 1.68 [1.43, 1.98] | .16 |
| CASP3 | 29.94 [10.76, 48.61] | 29.77 [19.33, 46.17] | .80 |
| uPA | 2.01 [1.34, 3.28] | 1.74 [1.10, 3.15] | .40 |
| CPB1 | 0.87 [0.79, 1.00] | 0.93 [0.82, 1.00] | .32 |
| CHI3L1 | 0.66 [0.40, 1.21] | 0.70 [0.34, 1.66] | .90 |
| ST2 | 2.16 [1.82, 2.54] | 2.33 [1.99, 2.77] | .18 |
| tPA | 2.59 [1.96, 3.15] | 2.16 [1.92, 3.13] | .24 |
| SCGB3A2 | 0.67 [0.61, 0.76] | 0.73 [0.70, 0.80] | .078 |
| EGFR | 0.95 [0.91, 0.99] | 0.97 [0.88, 1.00] | .99 |
| IGFBP7 | 48.69 [24.62, 100.28] | 48.20 [22.36, 81.88] | .82 |
| CD93 | 17.92 [11.16, 25.09] | 15.64 [10.70, 22.99] | .59 |
| IL18BP | 1.72 [1.39, 2.36] | 1.80 [1.56, 2.20] | .91 |
| COL1A1 | 1.09 [0.96, 1.46] | 1.02 [0.96, 1.10] | .054 |
| PON3 | 1.20 [1.09, 1.35] | 1.31 [1.21, 1.58] | .032 |
| CTSZ | 9.76 [5.81, 17.02] | 8.98 [4.13, 12.20] | .31 |
| MMP3 | 2.04 [1.58, 2.54] | 2.18 [1.79, 2.33] | .60 |
| RARRES2 | 4.35 [2.66, 8.49] | 4.98 [2.49, 9.61] | .98 |
| ICAM2 | 2.33 [2.15, 2.76] | 2.49 [2.26, 2.69] | .42 |
| KLK6 | 0.29 [0.21, 0.35] | 0.28 [0.24, 0.40] | .61 |
| PDGFsubunitA | 1.46 [1.37, 1.57] | 1.51 [1.33, 1.55] | .90 |
| TNFR1 | 7.20 [6.18, 9.18] | 6.95 [5.36, 8.11] | .30 |
| IGFBP2 | 9.55 [5.87, 14.66] | 8.27 [4.98, 11.58] | .41 |
| vWF | 2.00 [1.57, 2.52] | 2.12 [1.92, 2.40] | .25 |
| PECAM1 | 1.07 [0.90, 1.24] | 1.08 [0.91, 1.28] | .74 |
| MEPE | 1.36 [1.19, 1.59] | 1.39 [1.24, 1.69] | .61 |
| CCL16 | 0.82 [0.72, 0.98] | 0.85 [0.71, 1.07] | .79 |
