# Supplementary figures and images for: Comparison of cardiovascular biomarker expression in extracellular vesicles, plasma and carotid plaque for the prediction of MACE in CEA patients
Source: Sci Rep. 2023 Jan 18;13:1010. doi: 10.1038/s41598-023-27916-6 (PMC9849473; doi:10.1038/s41598-023-27916-6)

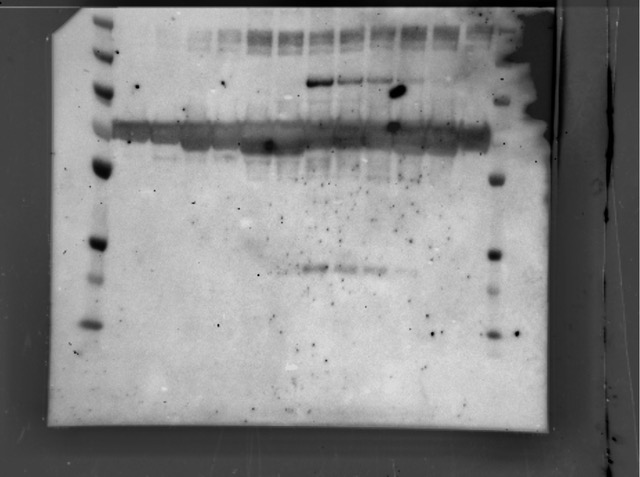

Supplement: Supplementary file 2 — Supplementary Figure S1. [file 41598_2023_27916_MOESM2_ESM.jpeg]

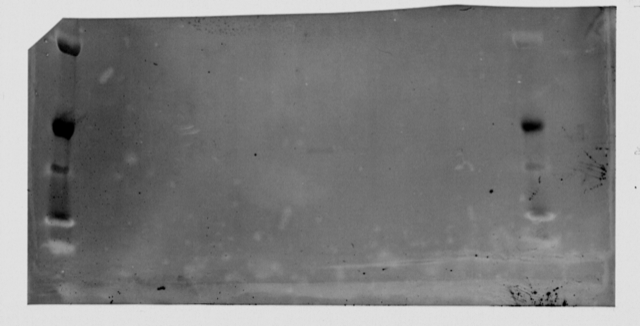

Supplement: Supplementary file 3 — Supplementary Figure S2. [file 41598_2023_27916_MOESM3_ESM.png]
